# CTSG may inhibit disease progression in HIV-related lung cancer patients by affecting immunosuppression

**DOI:** 10.1186/s13027-024-00599-y

**Published:** 2024-07-30

**Authors:** Xuan Yan, Shuoyan Wei, Yuexiang Yang, Zhangyan Zhao, Qingguo Wu, Haicheng Tang

**Affiliations:** 1 Department of Respiratory and Critical Care Medicine, Shanghai Public Health Clinical Center, Fudan University, No 2901, Caolang Road, Jinshan District, Shanghai, 201508 China; 2 Department of Pathology, Shanghai Public Health Clinical Center, Fudan University, Shanghai, China

**Keywords:** CTSG, Lung cancer, HIV infection, Immune microenvironment, Metastasis, Prognosis

## Abstract

**Objectives:**

Lung cancer is an independent risk factor for pulmonary complications following HIV infection. This study aimed to examine the expression and clinical significance of Cathepsin G (CTSG) protein in both non-HIV and HIV-related lung cancers.

**Methods:**

The data related to lung adenocarcinoma (LUAD) and lung squamous carcinoma (LUSC) in the TCGA dataset and the data related to healthy individuals in the GTEx dataset, the GEPIA2 database was used to excavate the distinction in the expression of CTSG protein in non-small cell lung cancer (NSCLC) tissues versus normal non-cancerous tissues. The Ualcan database was used to compare the differences in CTSG expression at different stages of LUAD and LUSC. Immunohistochemistry (IHC) was used to detect the expression of CTSG proteins in the pathological tissues of patients with HIV-related lung cancer and patients with lung cancer without co-infection, the Kaplan-Meier method was used for survival analysis.

**Results:**

We observed that CTSG expression in NSCLC is lower compared to adjacent non-tumor tissues and correlates with NSCLC clinical stage. CTSG protein expression in HIV-related lung cancer tissues was lower than in adjacent tissues and lower than in lung cancer tissues without HIV infection, with a statistically significant difference (*P* < 0.05). It correlated with CD4 + T cell count and CD4+/CD8 + T cell ratio, as well as with the pathological type, distant metastasis, and clinical stage of HIV-related lung cancer, all with statistical significance (*P* < 0.05).

**Conclusions:**

CTSG could potentially mitigate disease advancement in HIV-related lung cancer patients by inhibiting immune depletion, serving as a prospective immunotherapeutic target for both non-HIV and HIV-associated lung cancers.

## Introduction

As one of the leading causes of cancer-related death throughout the world, the development of lung cancer is closely linked to the role and regulation of the immune system [1]. In the past decade, the treatment paradigm of lung cancer has been greatly changed with intensive research on therapeutic strategies targeting immune checkpoint proteins. For the study of immune checkpoint proteins such as programmed death-1 (PD-1)/programmed death ligand 1 (PD-L1), immune checkpoint inhibitors (ICIs) have been validated as the first-line treatment for metastatic non-small cell lung cancer. However, the acquired resistance to ICIs in the treatment of lung cancer remains an unavoidable challenge [2]. Therefore, the cognitive process of lung cancer development and treatment still needs to be strengthened by the study of related immune system changes.

Pulmonary complications of human immunodeficiency virus-1 (HIV) have been a common cause of morbidity and mortality since its first discovery [3]. HIV can affect humoral and cellular immunity in the lungs in a number of aspects, with CD4 + T-lymphocytes being the main target cells of HIV, and low CD4+/CD8 + T cell ratio leading to the loss of functions of the respiratory system [4]. Lung cancer is an independent risk factor for poor prognosis in HIV-infected patients in advanced stages, and the weakened immune system in HIV patients in turn plays a contributory role in the development of lung cancer [5]. With the use of highly active antiretroviral therapy (HAART), lung cancer is becoming a major source of cancer deaths in people living with HIV (PLWH) [6]. In recent years, the prognosis of HIV-related lung cancer patients remains poor despite advances in anti-HIV treatment and comprehensive lung cancer treatment. Thus, exploring the immunological mechanism of lung cancer occurrence and development, and searching for new diagnostic markers and immune-related drug therapeutic targets are not only helpful for early diagnosis and prognosis judgment of lung cancer, but also of great significance for patients with HIV-related lung cancer to alleviate the negative impacts brought by malignant tumor, enhance immune function, and improve their survival in the presence of immune deficiencies.

Cathepsin G (CTSG), a member of the lysosome protein hydrolase family, is produced and secreted by a subpopulation of monocytes and activated neutrophils, and is a kind of intracellular protease present in most animal tissues. And the products of neutrophils may affect the characteristics of tumor cells, such as growth status, intercellular or extracellular matrix adhesion, etc. [7]. Not only that, Cathepsin G is also an immune-related regulatory molecule, identified as a chemotactic agent for monocytes and shown to stimulate lymphocyte proliferation [8, 9]. CTSG is involved in host immune activity, cleavage of inflammatory mediators, degradation of extracellular matrix components, and antigen presentation [10]. It has been demonstrated that CTSG has an inhibitory effect on colorectal cancer cells by controlling the activity of the anti-apoptotic signaling pathway, and CTSG is highly expressed in breast cancer cells [7, 11]. In addition, CTSG plays an important regulatory role in the development of LUSC [12]. At present, the expression, function and regulatory role of CTSG in HIV-related lung cancer tissues have not been reported. In this study, we are committed to excavating potent immune targets, and further explored the expression and clinical significance of CTSG in HIV-related lung cancer on the basis of a big data search for the expression and role of Cathepsin G in lung cancer.

## Materials and methods

### Data sources of CTSG expression in lung cancer

The gene expression data of tumor tissue and normal non-cancerous tissue of lung adenocarcinoma and lung squamous carcinoma patients were collected from the TCGA public dataset(https://www.cancer.gov/ccg/research/genome-sequencing/tcga) and the the GTEx dataset (https://gtexportal.org/home/), which included 486 tumor tissue data of LUSC patients and 338 normal non-cancer tissue data, 483 tumor tissue data of LUAD patients and 347 normal non-cancer tissue data. Clinical information and data of patients with LUAD or LUSC were obtained from the TCGA public dataset.

### Differential expression analysis of CTSG protein in lung cancer

The ANOVA algorithm in the GEPIA2 database [13] was utilized for differential gene expression analysis, and the screening condition was set as log2-fold change (log2FC) = 0.5 and the corresponding corrected *P* value < 0.01 to represent the expression difference. Plus, the expression levels of the target gene, CTSG, were compared between the tumor tissues and the normal non-cancerous tissues, and the expression levels of CTSG were also compared between the different clinical stages of LUAD and LUSC using the Ualcan database(https://ualcan.path.uab.edu/index.html).

### Analysis of the impact of CTSG on the survival of lung cancer patients

Survival analysis of screened LUAD and LUSC samples from TCGA public dataset was performed using the GEPIA2 database. Using the median CTSG gene expression in LUAD and LUSC samples as the threshold value (cut-off = 50%), the correlation between CTSG expression and overall survival as well as disease-free survival of LUAD and LUSC patients was analyzed by Log-rank test, and Kaplan-Meier survival curves were plotted.

### Study population and design

In this retrospective study, 45 HIV-related lung cancer tissues and corresponding adjacent non-tumor tissue specimens surgically resected at the cardio-thoracic surgery department of Shanghai Public Health Clinical Center (SPHCC), Shanghai, China from January 2020 to August 2023 were collected, and were placed in liquid nitrogen and transferred to a -80 °C refrigerator for storage. The diagnosis of the 45 patients was in accordance with the diagnostic protocol of HIV-related lung cancer. And all the patients had been diagnosed by pathological and histological examination, and had not received any form of preoperative treatments including chemoradiotherapy, targeted therapy and immunotherapy. And the basic clinical information and laboratory test indexes of 45 patients were also collected and analyzed (indicators collected were measured for the first time after the patient’s visit to the clinic). Inclusion criteria: age above 18 years old; patients with HIV confirmed by laboratory tests who were not on any treatment at the time of inclusion in the study; patients do not have lung cancer at the time of diagnosis of AIDS, and lung cancer occurs on the basis of the individual being an AIDS patient; patients participated voluntarily and signed informed consent. Exclusion criteria: patients with other tumors or autoimmune diseases.

Another 52 cases of simple lung cancer tissues resected in the same period were collected as a control group. The diagnosis of the 52 patients was in accordance with the diagnostic protocol of lung cancer. This study was approved by the ethics committee of the SPHCC (Ethics approval number: 2020-Y076-01), and was in strict conformity with the ethical guidelines of the Declaration of Helsinki. Written informed consent was obtained from all study participants.

### Immunohistochemistry (IHC)

Cathepsin G rabbit anti-human polyclonal antibody was purchased from GeneTex, and IHC SP kit and DAB chromogenic agen were purchased from Solarbio. Immunohistochemical staining was performed according to the instructions as follows: (1) Paraffin-embedded tissues are sliced using a machine, and then slices of approximately 4 micron thickness are selected and baked at 56 degrees for 2 h. (2) Dewaxing of slices, gradient rehydration, dimethylbenzene I: 5 min; dimethylbenzene II: 5 min; anhydrous ethanol: 5 min; 95% ethanol: 5 min; 85% ethanol: 5 min; distilled water: 5 min; PBS washing: 3 min for three times. (3) Microwave replication of antigens: the slices are heated with citrate buffer, the PH is adjusted to 6.0, and the temperature is brought to 100 degrees Celsius until deflated for 15 min, then the slices are removed and cooled slowly with cool water. (4) Slices were washed in distilled water and soaked in 0.3% H2O2 for 10 min and the slices were then washed with PBS for 5 min x 3 times. (5) Containment of non-specific antigens: Add a liquid containing 10% serum, put it into a wet box, and seal it at 37 degrees for 30 min. (6) Primary antibody incubation: dilute rabbit anti-human CTSG polyclonal antibody according to the ratio of 1:1000, cover each section with a volume of 100 µl, and incubate at 37℃ for 1 h or at 4℃ overnight. (7) Secondary antibody incubation: incubate at 37℃ for 40 min with secondary antibody, and then wash with PBS for 5 min×4 times. (8) DAB color development: DAB B solution 1mL plus C solution 20 µl for about 5 min (the substrate shows yellow), water rinse. (9) Hematoxylin stains nucleus for 2 min, 56 ℃ water rinse 1 min, respectively in 75%, 85%, 95% of the alcohol solution will be dehydrated for 5 min; after dehydration, in turn, with xylene I, xylene II transparent treatment, each need 5 min; the transparent slices were then baked in a constant temperature oven at 60℃ for 2 s. (10) Sealed with neutral gum to avoid air bubbles, and the sealed slices were placed flat for 24 h and dried for use.

### Immunohistochemical results quantification

The criteria for quantifying the immunohistochemical results of CTSG protein are mainly by observing the range and intensity of staining throughout the tissue specimen under light microscope. Positive expression of CTSG protein was mainly manifested as tan or brown granules on the cell membrane, and 2–3 different fields of view were taken at random. Cells with detectable CTSG expression on the cell membrane are positive cells. Quantification of CTSG expression on tissues was performed using the product of a score that calculates the percentage of positive cells and a score that calculates the strength of staining of positive cells. The percentage of positive cells (P) score criteria: 1 point is the percentage of positive cells ≤ 10%, 2 points is the percentage of positive cells > 10% and ≤ 50%, 3 points is the percentage of positive cells > 50% and ≤ 75%, and 4 points is the percentage of positive cells > 75%. At the same time, the criteria for the strength of positive cell staining score: 1 point for negative (-), 2 points for weakly stained (+), 3 points for moderately strongly stained (++), and 4 points for strongly stained (+++). Finally, the CTSG expression score of each tissue was obtained.

### Statistics

**Fig. 1:**
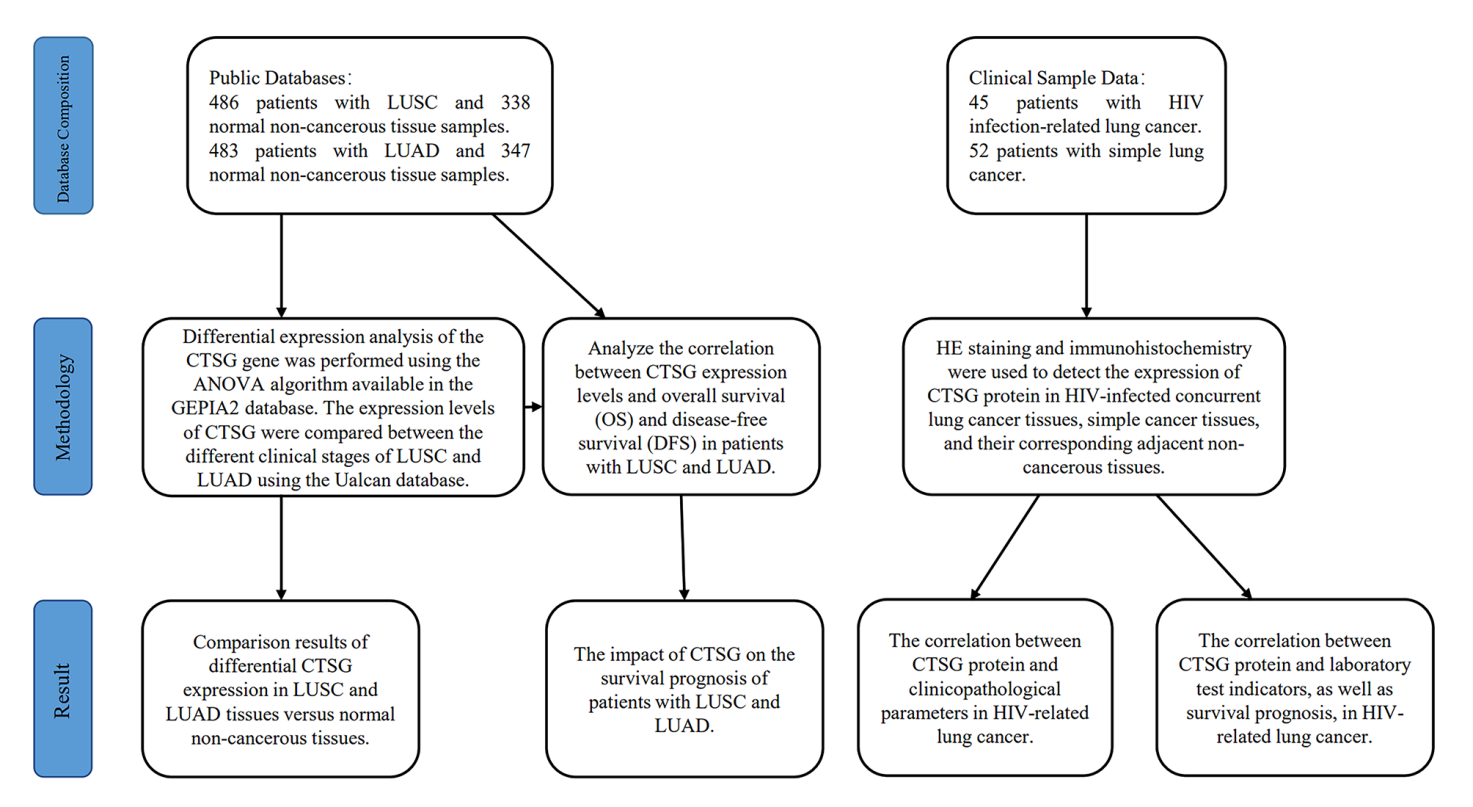
Experimental Flowchart for CTSG Protein Expression and Clinical Value in Lung Cancer and HIV-related Lung Cancer

The flow chart for the analysis process is shown in Fig. 1. Stata 16.0, GraphPad Prism 8.3.1 software was used for statistical analysis. Enumeration data was expressed as mean ± standard deviation (x ± s), and qualitative data was expressed as rate. Baseline characteristics are presented as mean (SD)for continuous variables. The χ2 test and t test were used to test statistical differences. Differences in proportions were evaluated usingχ2 tests. Log-rank test was used for survival prognosis analysis. All tests were two-sided, and a *P* value less than 0.05 was considered statistically significant.

## Results

### The expression of CTSG protein was different in non-small cell lung cancer tissues and normal non-cancer tissues

**Fig. 2:**
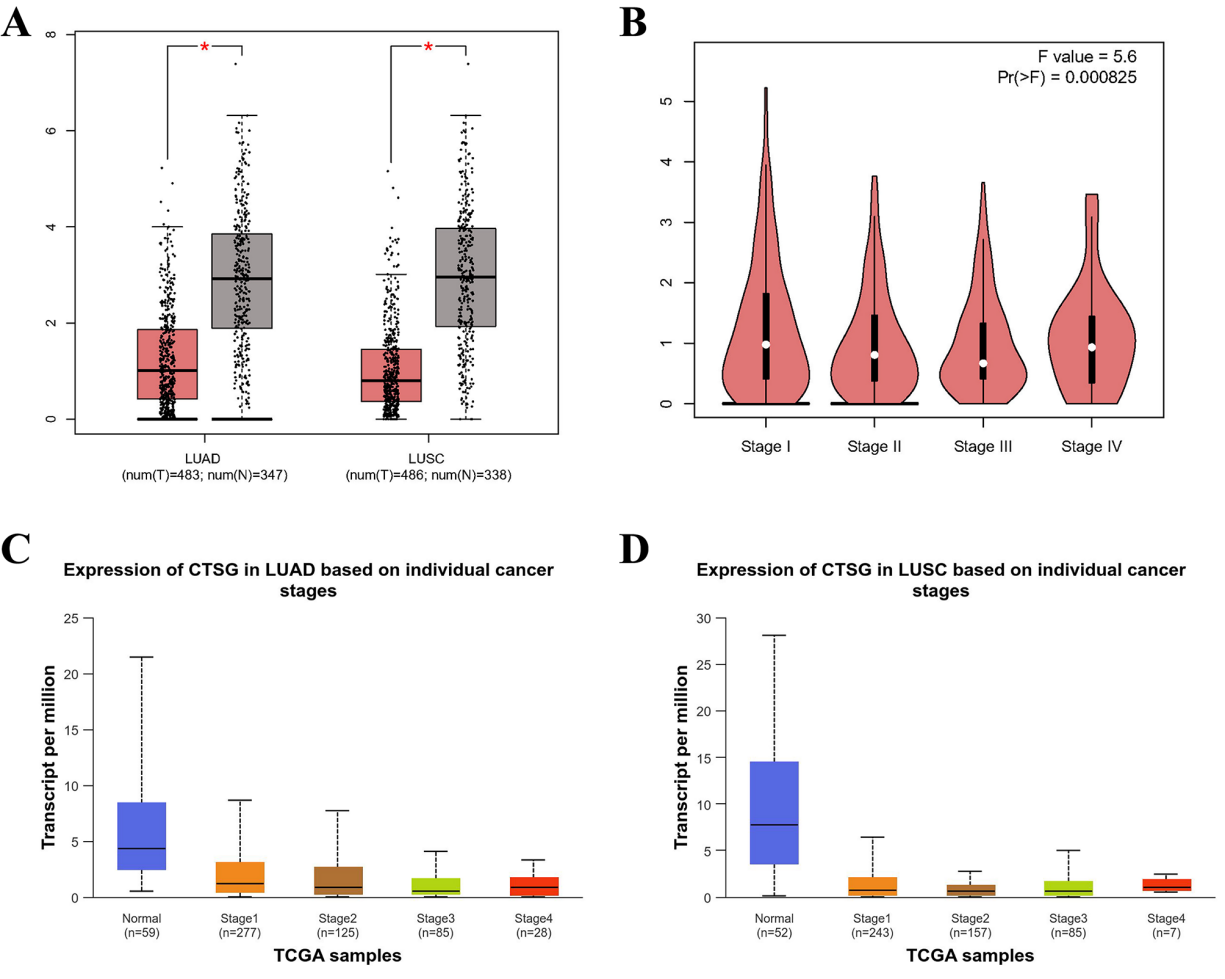
CTSG expression in non-small cell lung cancer. **A**: The expression difference of CTSG between tumor (red) and non-tumor (grey) tissues, * *P*<0.05; **B**: The expression difference of CTSG in different stages of LUAD and LUSC; **C**: Expression of CTSG in LUAD based on individual cancer stages; **D**: Expression of CTSG in LUSC based on individual cancer stages

Box plots were drawn based on the levels of CTSG expression in non-small cell lung cancer tissues and normal non-cancerous tissues (Fig. 2A), and the results demonstrated that the CTSG expression level in LUAD tissues (1.02) was significantly lower than that of normal non-cancerous tissues (6.57, log2FC =-1.906, *P* < 0. 01), and in LUSC tissues CTSG expression level (0.75) was significantly lower than that in normal non-cancerous tissues (6.74, log2FC =-2.149, *P* < 0. 01). In addition, the expression level of CTSG is significantly different in different stages of NSCLC (F = 5.6, *P* < 0. 01, Fig. 2B). Whether for LUAD or LUSC, the expression of CTSG in normal tissues was significantly higher than that in tumor tissues of different clinical stages (*P* < 0.05), and in clinical stages 1, 2, and 3, the more advanced the stage, the lower the expression of CTSG (Fig. 2C and D).

### Effect of CTSG on the prognosis of patients with non-small cell lung cancer

**Fig. 3:**
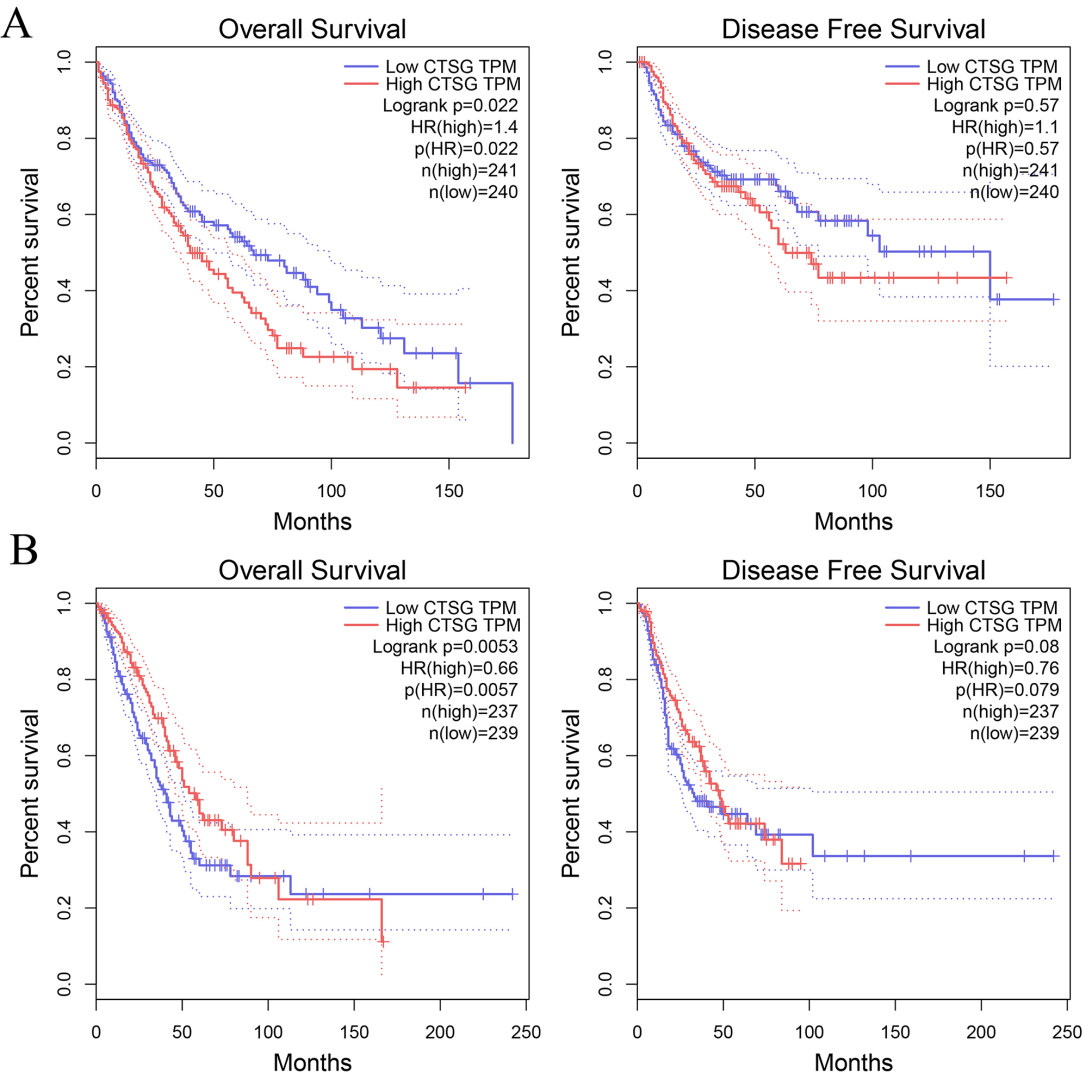
The impact of CTSG expression on overall survival and disease-free survival in patients with non-small cell lung cancer, **A**: Lung adenocarcinoma; **B**: Lung squamous carcinoma

According to the results of survival analysis, for both LUAD and LUSC patients with low CTSG transcripts per kilobase million (TPM), the overall survival (OS) was significantly shorter than that of those with high CTSG TPM (*P* < 0.05), but the level of CTSG TPM had no significant effect on the disease-free survival (DFS) of both LUAD and LUSC (*P* > 0.05) (Fig. 3). The results of the analysis predicted that the expression level of CTSG might have some influence on the survival prognosis of patients with non-small cell lung cancer.

### Baseline of participants

In our cohort, among the 45 HIV-related patients, 37 patients were male and 8 patients were female, the age of the patients ranged from 25 to 75 years old, with an average age of (60 ± 2) years old. There were 10 cases of LUSC, 31 cases of LUAD, and 4 cases of other pathologic types (including 3 cases of large cell carcinoma and 1case of carcinoid tumor). And 52 cases of simple lung cancer include 35 males and 17 females, and the patients’ age ranged from 40 to 86 years old, with an average age of (68 ± 1) years old. And there were 12 cases of LUSC and 40 cases of LUAD. In order to illustrate the differences in the expression of CTSG protein in different pathological tissues or normal tissues, we tried to collect cases with consistent indicators in baseline data when collecting samples from the two groups, so as to minimize the errors arising from the differences in individual clinical factors. The demographic and clinical characteristics of the 45 HIV-related patients and 52 simple lung cancer are shown in Table 1.

**Table 1:** Baseline of participants

	HIV-related Lung cancer (*n* = 45)	Simple Lung Cancer (*n* = 52)	*P*
Gender(male)	37/45 (82.22)	35/52 (67.31)	0.094
Age(years)	60 ± 2	68 ± 1	**< 0.001**
Smoking(yes)	11/45 (24.44)	14/52 (26.92)	0.781
Pathologic type of lung cancer (LUSC)	10/45 (22.22)	12/52 (23.08)	0.089
Pathologic type of lung cancer (LUAD)	31/45 (68.89)	40/52 (76.92)	0.089
Clinical staging (I / II/ III)	31/45 (68.89)	35/52 (67.31)	0.066
Lymph node metastasis (yes)	28/45 (62.22)	47/52 (90.38)	**0.001**
Distant metastasis	14/45 (31.11)	17/52 (32.69)	0.868
CD4 + T cell count(cell/µl) Mean value	322.96	522.75	**< 0.001**
CD8 + T cell count(cell/µl) Mean value	643.17	364.68	**< 0.001**
CD4 + T/CD8 + T cell count	0.69	1.92	**< 0.001**
CEA ^a^(ng/ml) Mean value	3.21	77.69	**0.018**
CA125 ^b^(U/ml) Mean value	29.95	50.28	0.099
ProGRP ^c^(pg/ml) Mean value	221.03	131.55	0.441
NSE ^d^(ng/ml) Mean value	11.70	14.83	0.294
SCC ^e^(ng/ml) Mean value	1.68	1.23	0.517
CYFRA21-1 ^f^(ng/ml) Mean value	4.16	10.98	**0.018**

*P* < 0.05 is considered statistically significant. a: The level of CEA was not measured in 3 patients with HIV-related Lung cancer. b: The level of CA125 was not measured in 3 patients with HIV-related Lung cancer. c: The level of ProGRP was not measured in 23 patients with HIV-related Lung cancer and 3 patients with simple lung cancer. d: The level of NSE was not measured in 8 patients with HIV-related Lung cancer. e: The level of SCC was not measured in 8 patients with HIV-related Lung cancer. f: The level of CYFRA21-1 was not measured in 8 patients with HIV-related Lung cancer

### Expression of CTSG proteins in HIV-related lung cancer tissues

**Fig. 4:**
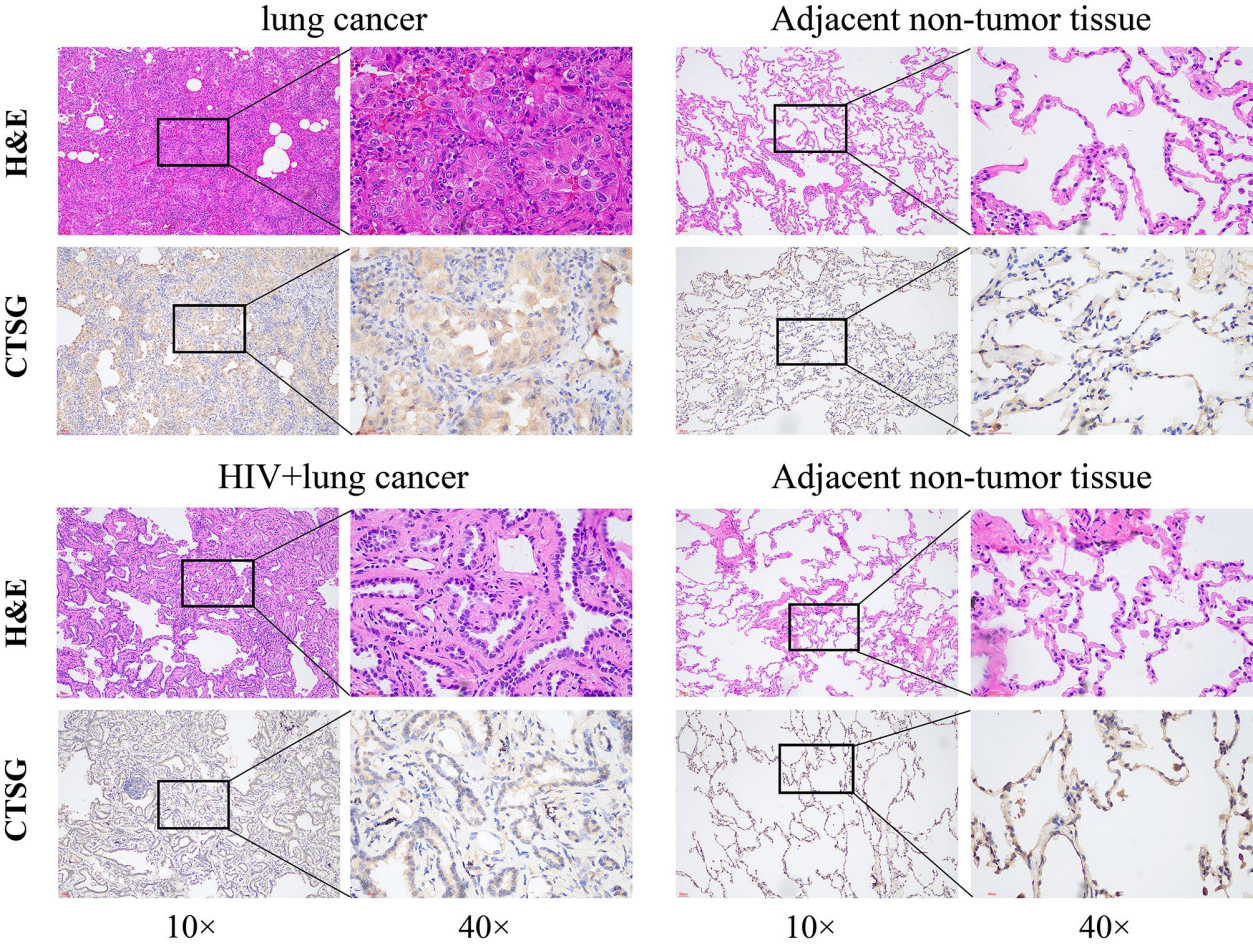
The expression of CTSG protein in simple lung cancer tissues, HIV-related lung cancer tissues and their corresponding adjacent non-tumor tissues detected by HE staining and IHC. (Scale bar = 50 μm)

Hematoxylin eosin (HE) staining and immunohistochemistry were used to detect the expression of CTSG protein in 45 cases of HIV-related lung cancer tissues, 52 cases of simple lung cancer tissues and their corresponding adjacent non-tumor tissues. The results showed that CTSG protein was mainly localized on the cell membrane and appeared as tan or brown granules. In non-small cell lung cancer, the expression of CTSG was lower than that in adjacent non-tumor tissues. Meanwhile, the expression of CTSG protein in HIV-related lung cancer tissues was not only significantly lower than that in adjacent non-tumor tissues, but also lower than that in simple non-small cell lung cancer tissues (Fig. 4). 40 of the 45 cases of HIV-related lung cancer tissues were positive for CTSG protein expression, with a positivity rate of 88.9%. The expression score of CTSG protein in 45 tissues was calculated according to the above CTSG immunohistochemical results quantification method, which resulted in the lowest score of CTSG expression in 45 tissues as 1 point, the highest as 18 points, and the average score was 7.8 points. Taking 7.8 points as the cut-off value, those > 7.8 and ≤ 18 points were considered as the high expression group, and those ≥ 1 and ≤ 7.8 points were considered as the low expression group, resulting in 20 cases in the low expression group and 25 cases in the high expression group, with a high expression rate of 55.6%. Additionally, the high CTSG expression rate of HIV-related LUAD was 74.19%, and the high expression rate of HIV-related LUSC was 10%, and the high CTSG expression rate of different pathologic type of HIV-related lung cancer was compared by using χ2 test, which shows that there is a difference in the CTSG expression of different pathologic types (*P*<0.05) (Table 2).

**Table 2:** Expression of CTSG protein in HIV-related non-small cell lung cancer and paraneoplastic tissues

Group	n	CTSG protein expression examples		High expression rate(%)	χ2	P
		Low level of expression	High level of expression			
Lung squamous cell carcinoma tissue	10	9	1	10.00	14.28	**0.001**
Lung adenocarcinoma tissue	31	8	23	74.19		
Other pathological tissues	4	3	1	25.00		

*P* < 0.05 is considered statistically significant

### Correlation between CTSG protein expression and clinicopathological parameters in HIV-related lung cancer

The statistical results of this experiment showed that the level of CTSG protein expression was not related to the sex, age, smoking history, whether HIV RNA could be detected after the first consultation, and lymph node metastasis of the patients with HIV-related lung cancer (*P*>0.05); however, it was closely related to the pathological type of HIV-related lung cancer, distant metastasis, and clinical stage, and the difference was statistically significant (*P*<0.05) (Table 3).

**Table 3:** Relationship between CTSG protein expression and clinicopathologic parameters in HIV-related lung cancer

	*n*(%)	CTSG protein expression examples		χ2	*P*
		Low level of expression	High level of expression		
Gender				1.490	0.222
Male	37(82)	18	19		
Female	8(18)	2	6		
Age				0.005	0.944
≤ 65	29(64)	13	16		
>65	16(36)	7	9		
Smoking				0.385	0.535
Yes	11(24)	4	7		
No	34(76)	16	18		
Clinical staging				4.500	**0.034**
I / II/ III	30(67)	10	20		
IV	15(33)	10	5		
Pathological type				14.279	**0.001**
Squamous cell carcinoma	10 (22)	9	1		
Adenocarcinoma	31(69)	8	23		
Other pathological tissues	4(9)	3	1		
Lymph node metastasis				0.118	0.731
Yes	28(62)	13	15		
No	17(38)	7	10		
Distant metastasis				5.993	**0.014**
Yes	14(31)	10	4		
No	31(69)	10	21		
First HIV RNA after consultation				0.076	0.783
Detected	17(38)	8	9		
Undetected	28(62)	12	16		

*P* < 0.05 is considered statistically significant

### Correlation of CTSG protein expression with laboratory test indicators and survival prognosis in HIV-related lung cancer

We collected laboratory test indicators in 45 patients with HIV-related lung cancer, which included CD4 + T cell count, CD8 + T cell count, CD4 + T/CD8 + T cell count, carcinoembryonic antigen (CEA), CA125, pro-gastrin releasing peptide (ProGRP), neuron-specific enolase (NSE), squamous cell carcinoma antigen (SCC), and cytokeratin 19 fragment (CYFRA21-1). These indicators are measured and collected at the patient’s first visit to the hospital.

**Fig. 5:**
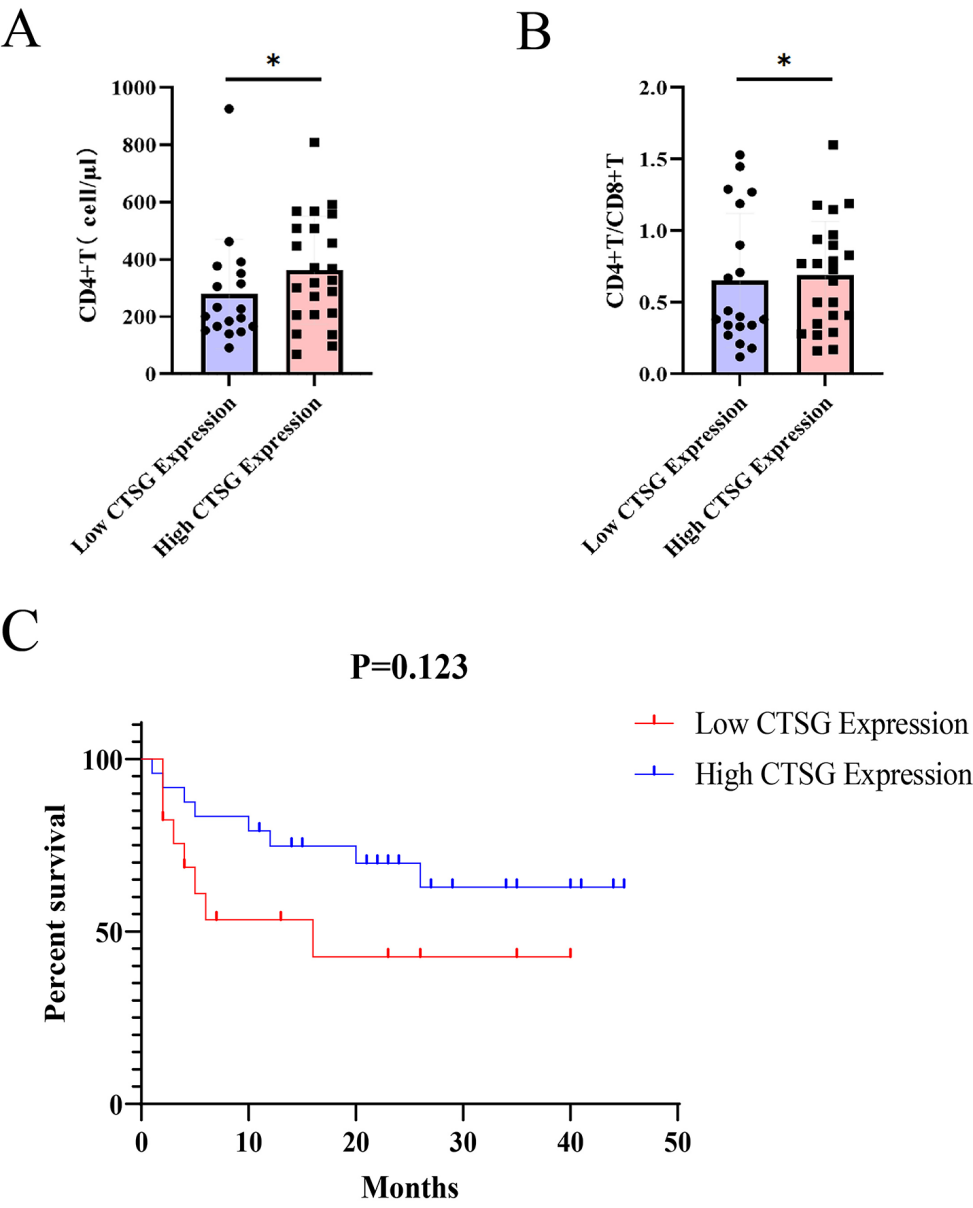
Correlation of CTSG protein expression level with laboratory test indicators and survival prognosis in HIV-related lung cancer, **A**: Statistics of the CD4 + T cell count in the CTSG high expression group and low expression group; **B**: Statistics of the CD4 + T/CD8 + T cells in the CTSG high expression group and low expression group; **C**: Survival curves of the CTSG high and low expression groups. *P < 0.05 by t-test

According to the immunohistochemical CTSG expression score of each pathological tissue, 45 samples were divided into high expression group and low expression group, and the t-test was used to compare the laboratory test indicators between the two groups, and the results showed that there were significant differences in the CD4 + T cell count and CD4 + T/CD8 + T cells between the two groups (*P* < 0.05). The mean value of CD4 + T cell count in the high CTSG expression group was higher than that in the low CTSG expression group (Fig. 5A). Plus, the mean value of CD4 + T/CD8 + T cells in the high CTSG expression group was higher than that in the low CTSG expression group (Fig. 5B). There was no significant difference between the high and low expression groups for the rest of the laboratory test indicators (Table 4).

**Table 4:** Relationship between CTSG protein expression and laboratory indicators in HIV-related lung cancer

	CTSG protein expression examples		t value	*P*
	Low level of expression	High level of expression		
CD4 + T cell count(cell/µl)			2.343	**0.024**
Mean value	250.68	384.83		
95%CIs	[177.91, 323.44]	[296.04, 473.62]		
CD8 + T cell count(cell/µl)			0.248	0.806
Mean value	627.70	655.54		
95% CIs	[481.37, 774.04]	[483.23, 827.85]		
CD4 + T/CD8 + T cell count			2.326	**0.025**
Mean value	0.515	0.801		
95% CIs	[0.35, 0.69]	[0.62, 0.99]		
CEA ^a^(ng/ml)			1.460	0.154
Mean value	2.610	3.876		
95% CIs	[1.36, 3.86]	[2.54, 5.21]		
CA125 ^b^(U/ml)			0.406	0.687
Mean value	31.786	21.394		
95% CIs	[14.08, 49.49]	[13.13, 41.66]		
ProGRP ^c^(pg/ml)			0.005	0.996
Mean value	222.162	220.581		
95% CIs	[212.43, 656.75]	[162.47, 693.63]		
NSE ^d^(ng/ml)			0.187	0.864
Mean value	11.975	11.513		
95% CIs	[8.51, 15 44]	[7.54, 15.48]		
SCC ^e^(ng/ml)			1.445	0.157
Mean value	2.856	0.881		
95% CIs	[0.67, 6.38]	[0.50, 1.27]		
CYFRA21-1 ^f^(ng/ml)			0.769	0.447
Mean value	3.586	4.560		
95% CIs	[1.96, 5.21]	[2.67, 6.45]		

*P* < 0.05 is considered statistically significant. a: The level of CEA was not measured in 3 patients. b: The level of CA125 was not measured in 3 patients. c: The level of ProGRP was not measured in 23 patients. d: The level of NSE was not measured in 8 patients. e: The level of SCC was not measured in 8 patients. f: The level of CYFRA21-1 was not measured in 8 patients

After a median follow-up of 15 (1–45) months, of the 45 patients with HIV-related lung cancer included in this study, 16 (35.6%) patients died after discharge from hospital and 4 (8.9%) patients were lost to follow-up. The shortest survival was 1 month and the longest survival was more than 45 months, with a 6-month survival rate of 72.1% (95% CIs: 0.55–0.83) and a 1-year survival rate of 66.4% (95% CIs: 0.4911–0.7898). All causes of death were thought to be related to HIV-related lung cancer. Grouped according to high and low CTSG expression, we performed a log-rank test to do the comparison of survival differences, which showed that the difference between the survival curves of the two groups was not statistically significant (*P* = 0.123). However, by plotting the survival curves, we could roughly see that the overall survival (OS) of the high CTSG expression group was slightly longer than that of the low CTSG expression group [HR(High) = 0.48], which was consistent with the trend that the overall survival (OS) of patients with low CTSG expression is significantly shorter than that of those with high CTSG expression in NSCLC retrieved by our big data (Fig. 5C).

## Discussion

With the development of antiretroviral therapy (ART), the life expectancy of HIV-positive patients has increased, while at the same time increasing the likelihood that they will suffer from complications of HIV infection, especially malignant tumors [14]. The incidence of lung cancer in HIV-positive patients is extremely significant, with two Meta-analyses showing that the standardized incidence ratios (SIRs) of lung cancer are more than 2.5 times higher in HIV-positive patients compared to the general population [15, 16]. Since the predominant pathogenesis in HIV-infected individuals is immunodeficiency, HIV infection can lead to a wide range of deficiency of humoral and cellular immunity functions, thereby increasing the risk of lung cancer. Based on the damage of HIV virus to the host immune system, together with the complex interactions between tumor cells, tumor immune microenvironment (TME) and host immunity, excavating more reliable predictive and therapeutic biomarkers is the key to improve the host immune function and immunotherapy for HIV-related lung cancer [2]. Through preliminary big data information retrieval, we found that the expression of Cathepsin G in lung cancer tissues was lower than that in non-tumor tissues, and the expression was different in different clinical stages and pathological types of lung cancer. By reviewing related studies, it is suggested that Cathepsin G is associated with the host immune regulation and shows a role in inhibiting the development of other types of cancer. Therefore, we collected HIV-related lung cancer specimens and simple lung cancer specimens, studied them by immunohistochemistry, and correlated them with the relationship between patients’ clinicopathological parameters, laboratory test indicators, and survival prognosis.

Cathepsins are proteolytic enzymes that are classified into several families, including serine proteases, cysteine proteases and aspartic proteases, etc. [17] There are about 15 kinds of Cathepsins in humans, and these enzymes are highly active and multifunctional in the lysosomal environment at low pH [18]. Among them, Cathepsin G is a kind of serine protease, which is one of the main components of azurophilic granules in neutrophils and can degrade microbial peptides. It plays an important role in eliminating intracellular and extracellular pathogens through non-oxidative pathways, decomposing tissues at sites of inflammation, and in anti-inflammatory responses. For instance, the granulomas that form after mycobacterium tuberculosis infects the lung are rich in Cathepsin G [18, 19]. 

During tumor invasion and metastasis, protein hydrolases play an important role in mediating the penetration of malignant tumor cells through the cell membrane. Among them, Cathepsin B and Cathepsin D may be prognostic markers of cancer [20]. The role of Cathepsin D in breast cancer was first reported in a study as early as 1980. This study found that hormone-sensitive breast cancer cell lines secreted a kind of estrogen-induced glycoprotein, which was later identified as a precursor of Cathepsin D [20]. Currently Cathepsin G has been less studied in tumors. It has been shown that Cathepsin G has a tumor-suppressive effect on colorectal cancer cells by controlling the activity of the Akt/mTOR/Bcl2-mediated anti-apoptotic signaling pathway [11]. And bioinformatics analysis showed that CTSG is a potential immune-related biomarker for oral squamous carcinoma [21]. Another study mentioned that the prognostic model of CTSG gene signatures was significantly associated with overall survival (OS) and tumor microenvironment (TME) immune cells in lung adenocarcinoma [12]. 

There are no clear findings and reports on the association and mechanism of CTSG either with lung cancer or HIV infection. However, several studies have suggested that CTSG may be related to the regulation of body immunity, and the TCGA public dataset and the GEPIA2 database have shown that the expression of CTSG protein in both LUSC and LUAD tissues is significantly lower than that in non-tumor tissues. We examined the expression of CTSG protein levels in surgical specimens from 45 patients with HIV-related lung cancer by immunohistochemistry, and found that CTSG proteins were mainly located in the cell membranes of lung cancer cells, and the analysis showed that the expression of CTSG proteins was significantly lower in HIV-related lung cancer tissues compared with adjacent non-tumor tissues. Not only that, we randomly collected tissues from 52 patients with simple lung cancer and used immunohistochemistry to detect their CTSG protein expression levels, which showed that the pathological tissues of patients with simple lung cancer had significantly higher CTSG expression than those of patients with HIV-related lung cancer, and the adjacent non-tumor tissues had significantly higher CTSG expression than the cancerous tissues, which is in line with the large data that showed that in non-small-cell lung cancer tissues, CTSG expression was significantly lower than that in non-tumor tissues.

Moreover, we divided HIV-related lung cancer patients into high expression group and low expression group according to the average level of CTSG protein expression in pathological tissues, and analyzed the relationship between CTSG protein expression and clinicopathological parameters by stratified analysis. The results showed that CTSG protein expression was not associated with sex, age, smoking history, lymph node metastasis and whether HIV viral RNA was detected after the first consultation (*P*>0.05), but was closely related to the pathological type, distant metastasis and clinical stage, with a statistically significant difference (*P*<0.05). The results of the follow-up of HIV-related lung cancer patients were compiled and log-rank test was applied, and the results showed that there was no significant difference in overall survival (OS) between the high and low CTSG protein expression groups, but the Kaplan-Meier survival curves plotted showed a trend of longer OS in the group with high expression of CTSG protein, which is consistent with the Kaplan-Meier survival curve of the impact of CTSG on the survival of non-small cell lung cancer patients in the database, and the expression of CTSG protein may also be a possible predictor of prognosis of patients with simple lung cancers as well as those with HIV-related lung cancers. In addition, from the collected laboratory test data, it was found that there was a significant difference between the CTSG high and low expression groups in terms of CD4 + T cell count and CD4 + T/CD8 + T cells, with the high expression group having a higher CD4 + T cell count and CD4 + T/CD8 + T cells. It is hypothesized that CTSG is a protective factor in the development of non-small cell lung cancer and HIV-related lung cancer, delaying the immune damage caused by HIV virus to the organism and the development of lung cancer; low expression of CTSG protein promotes the development of non-small cell lung cancer and HIV-related lung cancer, which may suggest that patients have a poor prognosis.

There have been clear studies confirming that CTSG molecules play an important role in clearing pathogens and counteracting inflammatory responses, but there are no definitive studies on CTSG’s role in anti-tumor and promoting immune function.

CTSG in serum has been reported to induce protease-activated receptor 2 (PAR2) expression and alter the cytoskeleton of human dermal microvascular endothelial cells [22]. Another study suggested that CTSG modulates inflammatory responses, activates matrix metalloproteinases (MMPs), and promotes coagulation [23]. It is thus promising to see whether CTSG proteins, which are highly expressed in cancerous tissues, can inhibit lung cancer progression through the modification of tumor microvasculature and the regulation of tumor microenvironment (TME). Whether CTSG can affect immune cells and immune-related molecules in immunodeficient patients in a direct or indirect manner is likewise a topic of interest. Future studies of CTSG-mediated novel HIV-related lung cancer treatment regimens may significantly improve the life span and quality of life of patients with HIV-related lung cancer.

## Conclusions

Our study showed that CTSG protein was lowly expressed in non-small cell lung cancer and the expression correlated with the clinical stage. CTSG expression in HIV-related lung cancer tissues was lower than in the adjacent non-tumor tissues and lower than in simple lung cancer. CTSG protein correlates with CD4 + T cell count, CD4 + T/CD8 + T cells, pathological type, distant metastasis and clinical stage in HIV-related lung cancer. Cathepsin G may be valuable for the diagnosis, efficacy judgment and prognosis assessment of non-small cell lung cancer and HIV-related lung cancer, but its related molecular mechanism needs to be further explored in multi-center experimental and clinical studies, which is expected to provide a theoretical basis for the treatment of non-small cell lung cancer and HIV-related lung cancer with CTSG as the target.

## Data Availability

No datasets were generated or analysed during the current study.
